# First-Principles Study of Vacancies in Ti_3_SiC_2_ and Ti_3_AlC_2_

**DOI:** 10.3390/ma10020103

**Published:** 2017-01-25

**Authors:** Hui Wang, Han Han, Gen Yin, Chang-Ying Wang, Yu-Yang Hou, Jun Tang, Jian-Xing Dai, Cui-Lan Ren, Wei Zhang, Ping Huai

**Affiliations:** 1 School of Physics and Engineering, Henan University of Science and Technology, Luoyang 471003, China; nkxirainbow@gmail.com (H.W.); hyy9539@163.com (Y.-Y.H.); Tangjunguyue@126.com (J.T.); 2 Shanghai Institute of Applied Physics, Chinese Academy of Sciences, Shanghai 201800, China; wangchangying@sinap.ac.cn (C.-Y.W.); daijianxing@sinap.ac.cn (J.-X.D.); rencuilan@sinap.ac.cn (C.-L.R.); zhangwei@sinap.ac.cn (W.Z.); 3 Department of Electrical Engineering, University of California, Los Angeles, CA 90095, USA; genyin@ucla.edu

**Keywords:** MAX phases, vacancies, diffusion barrier, density functional theory

## Abstract

MAX phase materials have attracted increased attention due to their unique combination of ceramic and metallic properties. In this study, the properties of vacancies in Ti_3_AlC_2_ and Ti_3_SiC_2_, which are two of the most widely studied MAX phases, were investigated using first-principles calculations. Our calculations indicate that the stabilities of vacancies in Ti_3_SiC_2_ and Ti_3_AlC_2_ differ greatly from those previously reported for Cr_2_AlC. The order of the formation energies of vacancies is V_Ti(a)_ > V_Ti(b)_ > V_C_ > V_A_ for both Ti_3_SiC_2_ and Ti_3_AlC_2_. Although the diffusion barriers for Ti_3_SiC_2_ and Ti_3_AlC_2_ are similar (~0.95 eV), the properties of their vacancies are significantly different. Our results show that the vacancy–vacancy interaction is attractive in Ti_3_AlC_2_ but repulsive in Ti_3_SiC_2_. The introduction of V_Ti_ and V_C_ vacancies results in the lattice constant *c* along the [0001] direction increasing for both Ti_3_SiC_2_ and Ti_3_AlC_2_. In contrast, the lattice constant *c* decreases significantly when V_A_ are introduced. The different effect of V_A_ on the lattice constants is explained by enhanced interactions of nearby Ti layers.

## 1. Introduction

The MAX phases form a large family of ternary carbides/nitrides with the general formula Mn+1AXn, where n varies from 1 to 3, M is an early transition metal, A is an A-group element, and X is C or N [1,2,3]. The MAX phases have a unique combination of the properties of ceramics and metals. Similar to metals, they are electrically and thermally conductive, easy to machine, ductile at high temperatures, and exceptionally resistant to damage and thermal shock. Like ceramics, they are elastically rigid, lightweight, and oxidation resistant.

Titanium aluminum carbide (Ti_3_AlC_2_) and titanium silicon carbide (Ti_3_SiC_2_) are 312 MAX phases. Like most MAX phases, Ti_3_SiC_2_ is stiff (Young’s modulus 352 GPa [4], tough (toughness 9 MPa m^1/2^ [5]), thermally conductive (37 W·m^−1^·K^−1^ [6]), and electrically conductive (4.5 × 10^6^ Ω^−1^·m^−1^ [7]). The properties of Ti_3_AlC_2_ differ slightly from those of Ti_3_SiC_2_; for example, it has a lower Young’s modulus (297 GPa) and electrical conductivity (2.9 × 10^6^ Ω^−1^·m^−1^). Both materials are readily machinable and tolerant to damage and thermal shock [8]. Ti_3_SiC_2_, has two polymorphs, i.e., α and β phases [9]. α-Ti_3_SiC_2_ has the same structure as Ti_3_AlC_2_, but the Si layer in β-Ti_3_SiC_2_ is shifted. β-Ti_3_SiC_2_ is a metastable phase, therefore α-Ti_3_SiC_2_ has been more widely studied [8,10]. For simplicity, only α-Ti_3_SiC_2_ is considered in this work.

On one hand, defects can be unintentionally introduced into Ti_3_SiC_2_ and Ti_3_AlC_2_ during their synthesis. These materials are refractory ceramics and considerable concentrations of vacancies and impurities are introduced during their multi-component nanolaminate formation. On the other hand, Ti_3_SiC_2_ and Ti_3_AlC_2_ are potential structural materials for nuclear applications. Defects are created in the lattices of Ti_3_SiC_2_ and Ti_3_AlC_2_ by irradiation when the incident particles displace atoms from their substitutional positions. As mentioned above, their unique properties make Ti_3_SiC_2_ and Ti_3_AlC_2_ suitable candidates to be adopted in applications where materials are subject to extreme environments, such as nuclear reactors [11,12,13,14]. Amorphization is an important factor to evaluate the irradiation-resistant of a material. The resistance of amorphization is dependent on the competing effects between the defect production and annihilation rate. Vacancies are the simplest defects in MAX phases. A deeper knowledge of the properties of them in Ti_3_SiC_2_ and Ti_3_AlC_2_ is crucial for the understanding of the defect production, annihilation process, and phase stability [15,16,17,18,19,20,21,22,23].

A large number of experimental studies [11,12,13,14,24,25,26,27,28,29,30,31] have investigated the properties of Ti_3_SiC_2_ and Ti_3_AlC_2_ when subjected to irradiation with heavy ions and neutrons. Nappé et al. [11] studied the defect properties of Ti_3_SiC_2_ under Au-, Kr-, and Xe-ion irradiation. They observed generation of many defects in the structure and an expansion along the *c* axis. Liu et al. [12] reported that atomic disorder appeared in Ti_3_(Si,Al)C_2_ after Kr-ion irradiation, but the typical layered structure was preserved. Whittle et al. [13] reported that Ti_3_AlC_2_ and Ti_3_SiC_2_ showed high tolerance to damage from Xe-ion irradiation. Hoffman et al. [14] compared the neutron irradiation tolerances of Ti_3_SiC_2_ and Ti_3_AlC_2_ with those of SiC and alloy 617. They concluded that Ti_3_SiC_2_ and Ti_3_AlC_2_ have good irradiation tolerances.

Compared with experimental methods, first-principles calculations have the advantage of enabling the study of materials at the atomic scale. Such calculations have frequently been used to predict the crystal structures and stabilities of MAX phases, and to model their defects and related properties. Wang et al. [15,16,17] systematically studied the effects of vacancies and impurities in the Ti_2_AlC phase. They calculated the stabilities of Ti_2_AlC samples with different types of vacancies. Music et al. [18] studied the vacancies in Ti_4_AlN_3_, and reported that the introduction of about 25% N vacancies in Ti_4_AlN_3_ is energetically favorable. Tan et al. [19] studied vacancy diffusion in Ti_2_AlC and its impurity phase Ti_3_AlC. Du et al. [20] studied the C vacancies in Ta_4_AlC_3_, and suggested that the introducing of C vacancies decreases the phase stability. Han et al. [21] studied defect stabilities in Cr_2_AlC under different magnetic orderings.

However, although Ti_3_SiC_2_ and Ti_3_AlC_2_ are the most extensively studied MAX phases experimentally, theoretical investigations of their defect properties are rare. Medvedev at al. [22] studied the influence of disorder associated with the presence of vacancies on the electronic properties of Ti_3_SiC_2_. They found that the presence of C vacancies in Ti_3_SiC_2_ caused local perturbations of the electronic structures. Zhao et al. [23] studied the formation energies of different defects in Ti_3_SiC_2_ and Ti_3_AlC_2_. They found that replacement of Ti by Al in Ti_3_AlC_2_ was more energetically favorable than replacement of Ti by Si in Ti_3_SiC_2_. These previous theoretical works mainly investigated point defect stabilities. In this work, we focus on the formation, stability, geometry, and diffusion properties of vacancies in Ti_3_SiC_2_ and Ti_3_AlC_2_.

## 2. Theoretical Method

Our calculations were performed under the framework of density functional theory as implemented in the Vienna ab initio simulation package (VASP) [32,33]. The projected augmented wave method (PAW) [34] and the generalized gradient approximation (GGA) [35] were used. According to our previous study on MAX phases [19,21], the exchange and correlation energies were calculated using the Perdew−Burke−Ernzerhof (PBE) functional [36]. The wave functions were expanded in a plane-wave basis set with an energy cutoff of 400 eV. The lattice constants and internal freedom of the unit cell were fully optimized until the Hellman-Feynman forces on the atoms were less than 0.01 eV/Å. The effective charge for each atom (charge difference after bonding) is given using Bader charge analysis [37].

In order to simulate a single vacancy structure, we employed a 2 × 2 × 1 supercell, which contains 48 atoms. According to our previous studies on defects properties of MAX phases [19,21,38], the supercell has been proved to be sufficient to reproduce the defect structures. The special k-point sampling integration was used over the Brillouin zone by using the Γ-centered 5 × 5 × 5 for this supercell [39]. All these calculation setups were checked using a larger energy cutoff and k-mesh; the results of total energy and Hellmann-Feynman forces are convergent within 0.01 eV and 0.01 eV/Å, respectively.

To evaluate the energy barrier of an Al-vacancy, the climbing image nudged elastic band method (cNEB) [40,41] was employed to investigate the saddle points and minimum energy paths for vacancy diffusion from the initial state to the final state. In all transition state search calculations performed in this paper, a total of eight images were used (not including the initial and final images of each transition).

## 3. Results and Discussion

### 3.1. Properties of Perfect Ti_3_SiC_2_ and Ti_3_AlC_2_

**Figure 1:**
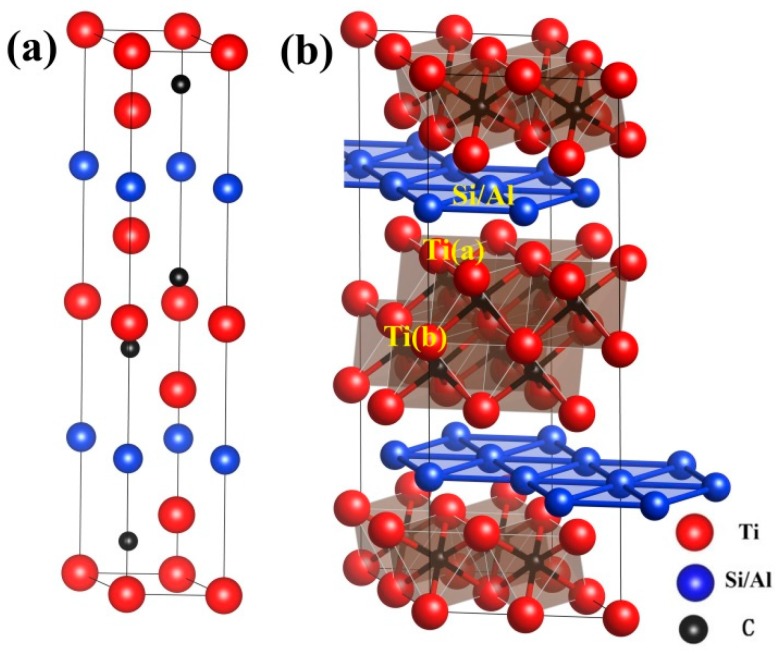
Crystal structures of Ti_3_SiC_2_ and Ti_3_AlC_2_: (**a**) conventional cell and (**b**) supercell used to model defect configurations. Red, blue, and black balls represent Ti, Si/Al, and C atoms, respectively. Two types of non-equivalent Ti atoms are identified.

Ti_3_SiC_2_ and Ti_3_AlC_2_ are both belonging to 312 phases with the same crystal symmetries, as shown in Figure 1. They are based on layers of hexagonally close-packed Ti and Al/Si layers with C occupying octahedral centers between the Ti layers. The structures of Ti_3_SiC_2_ and Ti_3_AlC_2_ can also be regarded as alternating stacks of two layers of edge-sharing Ti_6_C octahedra and a planar close-packed Al/Si layer. The Si/Al atoms are located in the Wyckoff 2*b* (0, 0, 1/4) positions and the C atoms are in *4f* (1/3, 2/3, *z*_C_) positions. There are two types of non-equivalent Ti atoms, denoted by Ti(a) and Ti(b), which are located at 4*f* (1/3, 2/3, *z*_Ti_) and 2*a* (0, 0, 0), respectively. The calculated structural parameters for Ti_3_SiC_2_ and Ti_3_AlC_2_ are listed in Table 1; the experimental results are also listed for comparison. The differences between the calculated and experimental values of the lattice constants are all smaller than 1%, indicating reliable predictions by our PBE calculations.

After optimization of the crystal structures, the mechanical property parameters were calculated. In the Voigt–Reuss–Hill approximation [43,44,45], the bulk modulus *B*, and the shear modulus *G* are the average of the values obtained by Voigt and Reuss approximations [43]. The Young’s modulus (*E*), the Poisson ratio (*v*), the transverse (*V_t_*), longitudinal (*V_l_*), and average (*V_a_*) acoustic wave velocities, and the Debye temperature (*Θ*_D_) can be obtained. Experimental values for Ti_3_AlC_2_ have not been reported, therefore only the calculated values for Ti_3_SiC_2_ are listed in Table 2 and compared with the experimental values. The results show that the calculated values are reasonably consistent with the experimental results. 

### 3.2. Formation Energies of Vacancies in Ti_3_SiC_2_ and Ti_3_AlC_2_

The stabilities of vacancies at different atomic sites in crystals can be evaluated by the vacancy formation energy, which is defined as follows:

*E*_vac_(V_X_) = *E*_tot_(V_X_) − *E*_tot_(perf) + *μ*_X_, (1)

where *E*_vac_(V_X_) is the vacancy formation energy of atom X (X = Ti, Al, C), *E*_tot_(V_X_) is the calculated total energy of a cell with defect X, *E*_tot_(perf) is the total energy of a perfect crystal without defects, and *μ*_X_ is the chemical potential of X. Here, *μ*_X_ is chosen as the energy of an isolated X atom for simplicity.

**Figure 2:**
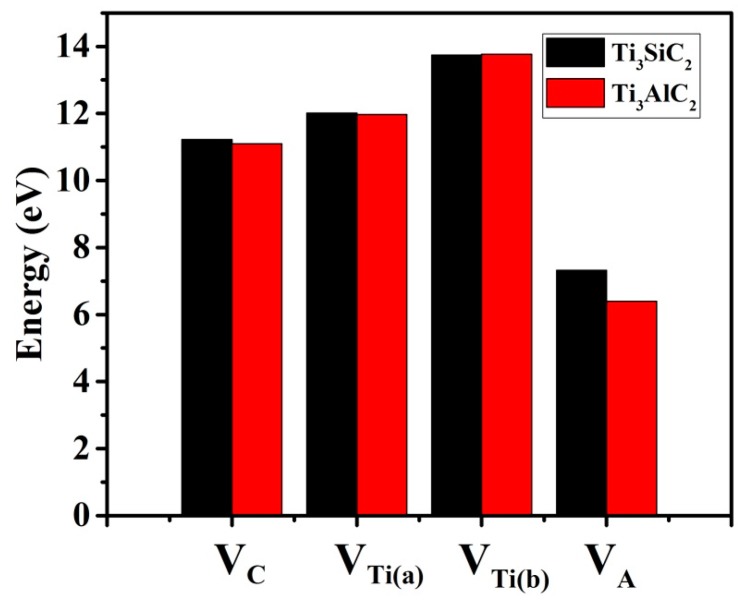
Vacancy formation energies (*E*_vac_, eV) of V_C_, V_Ti(a)_, V_Ti(b)_, and V_A_ (V_Si_/V_Al_) in Ti_3_SiC_2_ and Ti_3_AlC_2_.

As shown in Figure 2, for both Ti_3_SiC_2_ and Ti_3_AlC_2_, A-group element vacancies have the lowest formation energies, indicating that they are easily formed. The non-equivalent Ti(a) and Ti(b) atoms have different vacancy formation energies. Figure 1 shows that the Ti(a) atoms are located between Al and C layers. Ti(a) forms covalent bonds with C atoms, but forms weak metallic bonds with Al atoms. In contrast, the Ti(b) atoms are located at the center of [Ti_6_C] octahedra, and have stronger interactions with surrounding atoms. The vacancy formation energies of the Ti(b) atoms are therefore larger than those of the Ti(a) atoms. The order of the vacancy formation energies is V_Ti(a)_ > V_Ti(b)_ > V_C_ > V_A_. These results for Ti_3_SiC_2_ and Ti_3_AlC_2_ differ greatly from our previously reported results for Cr_2_AlC, in which the Al vacancies were predicted to have high formation energies and the Cr vacancies were predicted to have low formation energies [21]. The formation energy of V_Al_ is 0.9 eV lower than that of V_Si_, indicating that an A-group element mono-vacancy is more easily formed in Ti_3_AlC_2_.

### 3.3. Vacancy–Vacancy Interactions of V_A_

**Figure 3:**
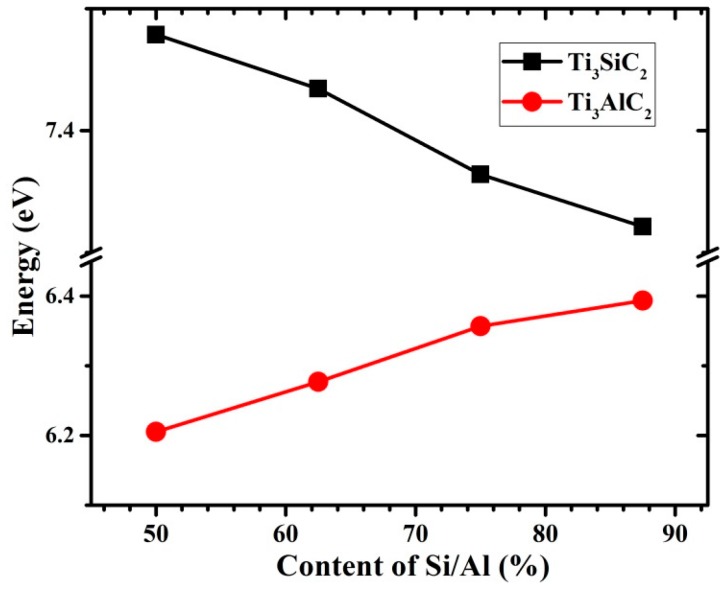
Vacancy formation energy (*E*_vac_, eV) of V_A_ (V_Si_/V_Al_) dependences on A-group element atomic content of Ti_3_SiC_2_ and Ti_3_AlC_2_.

These above calculation results indicate that V_A_ vacancies are easily formed when Ti_3_SiC_2_ and Ti_3_AlC_2_ are in oxidizing, corrosive, and irradiation environments. The effects of V_A_ vacancies on the phase stabilities of Ti_3_SiC_2_ and Ti_3_AlC_2_ were explored by introducing more vacancies and calculating their vacancy formation energies:

*E*_vac_(V_X_) = *E*_tot_(V_X_) − *E*_tot_(perf) + *μ*_X_, (2)

where *n* is the concentration of V_A_ vacancies in Ti_3_SiC_2_ and Ti_3_AlC_2_. Figure 3 shows that for Ti_3_AlC_2_ the vacancy formation energy decreases as the number of vacancies increases, indicating that existing vacancies can accelerate the formation of new vacancies. Therefore, decomposition of Ti_3_AlC_2_ can be caused by formation of a large number of vacancies in the Al layers. In contrast, the relationship between the V_A_ content and the vacancy formation energy is different for Ti_3_SiC_2_; the vacancy formation energy increases significantly with the increasing number of vacancies. This indicates that it is difficult to introduce a new V_Si_ near the original one because of the increased vacancy formation energy. Based on these results, it is reasonable to conclude that the interactions between nearby vacancies in Ti_3_AlC_2_ are attractive, but are repulsive in Ti_3_SiC_2_. The vacancies therefore tend to disperse in Ti_3_SiC_2_ but are accommodated in Ti_3_AlC_2_.

**Figure 4:**
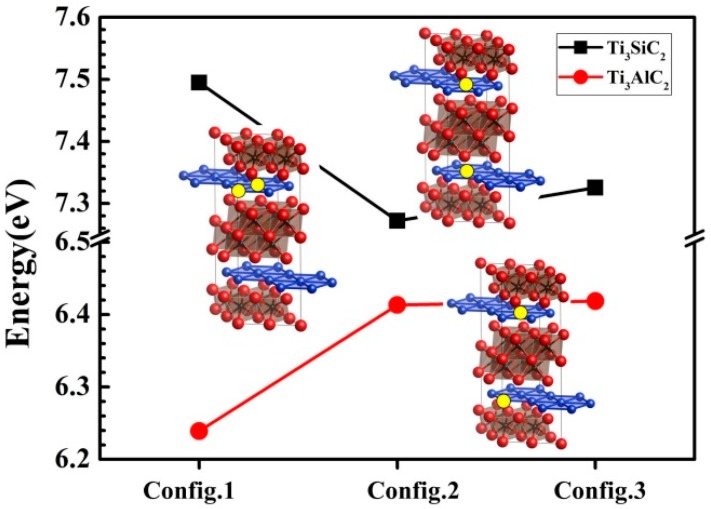
Vacancy formation energies (*E*_vac_, eV) for V_A_ (V_Si_/V_Al_) in three defect configurations of Ti_3_SiC_2_ and Ti_3_AlC_2_. Yellow circles represent vacancies in Si/Al layers.

In order to verify this conclusion, we calculated and compared the vacancy formation energies for three configurations with two vacancies introduced at different locations. The results are shown in Figure 4. For Ti_3_SiC_2_, the configuration with two vacancies located in different layers has a low formation energy. Vacancy pair formation (config.1) increases the energy by ~0.2 eV compared with the other two configurations (config.2 and config.3). In contrast, config.1 is energetically more favorable for Ti_3_AlC_2_. Therefore, Ti_3_SiC_2_ should be more stable than Ti_3_AlC_2_ in a corrosive environment.

### 3.4. Diffusion of V_A_ Vacancies

It is well known that the Al/Si atoms move in MAX phases predominantly by vacancy-mediated diffusion [19,21]. To ensure that the supercell was sufficiently large to avoid the influence of adjacent cells, a √3 × 2√3 × 1 supercell was used to calculate the diffusion barrier. The obtained values are consistent with those obtained using a 2 × 2 × 1 supercell. 

**Figure 5:**
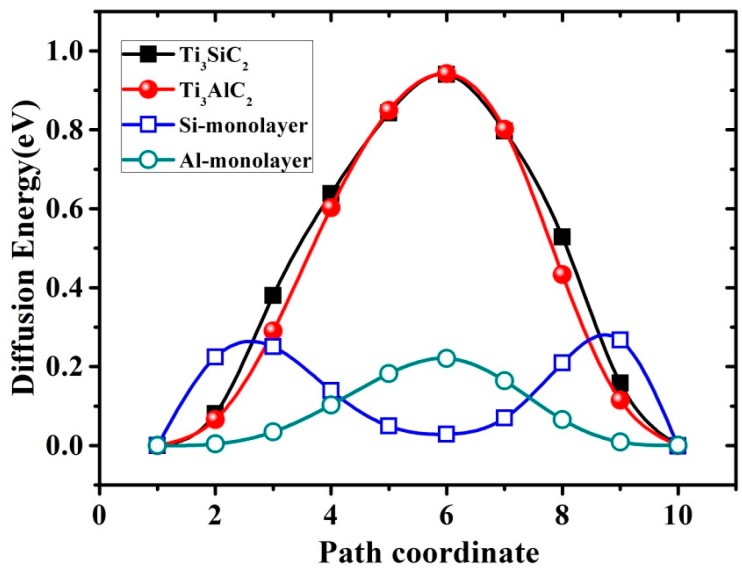
Calculated energy plots for diffusion of Si/Al vacancy in Ti_3_SiC_2_ and Ti_3_AlC_2_ using cNEB method. Energy barriers (*B*_diff_) for Ti_3_SiC_2_ and Ti_3_AlC_2_ are both 0.95 eV. Empty squares/circles denote energies for Si/Al vacancy diffusion in free-standing Si/Al layers; these indicate a low barrier of ~0.2 eV.

The calculated diffusion barriers (*B*_diff_) for Si/Al in Ti_3_SiC_2_ and Ti_3_AlC_2_ are less than 1 eV; these are close to the self-diffusion barriers of many metals, as shown in Table 3. The diffusion of vacancies along the (0001) plane can therefore occur frequently in these two materials. As mentioned previously, the interactions of vacancies in Ti_3_SiC_2_ are repulsive, whereas they are attractive in Ti_3_AlC_2_. A new vacancy will therefore diffuse away from an existing vacancy in Ti_3_SiC_2_; this does not greatly affect the stability of the material. In contrast, the low diffusion barrier indicates that vacancies in Ti_3_AlC_2_ tend to be accommodated. A large number of vacancies may therefore lead to decomposition of the material. The diffusion of atoms in the corresponding free-standing Si/Al layers was also studied using the same method shown in Figure 5. The diffusion barriers in free-standing layers (~0.2 eV) are clearly different from those in the Si/Al layers of MAX phases (~0.95 eV). These results indicate that the main contribution to the barrier is the interaction between the Al/Si and Ti layers, rather than the interaction in the Si/Al layers.

### 3.5. Effects of Vacancies on Lattice Constants

**Figure 6:**
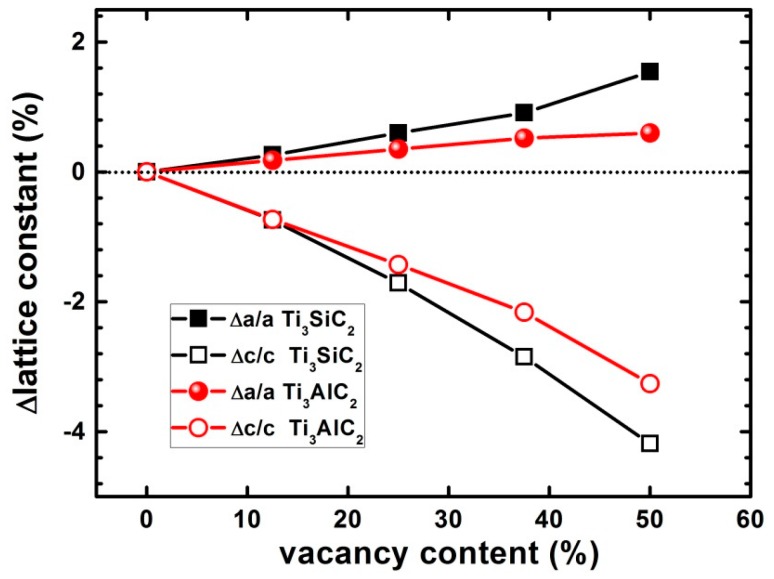
Changes in lattice constants of Ti_3_SiC_2_ and Ti_3_AlC_2_ with respect to concentration of Si/Al vacancies. Black and red lines indicate results for Ti_3_SiC_2_ and Ti_3_AlC_2_, respectively.

Defects in a material can lead to changes in the lattice constants. For example, irradiation of nuclear graphite increases the lattice constant *c* along the [0001] direction, and decreases the lattice constants *a* and *b* in the (0001) plane. This is because of the large numbers of interstitial carbons in the graphite interlayers. In this work, the effects of vacancies on the lattice constants of Ti_3_SiC_2_ and Ti_3_AlC_2_ were investigated. Figure 6 shows the trends in the changes in the lattice constants of Ti_3_SiC_2_ and Ti_3_AlC_2_ with increasing the number of vacancies in the supercell. The introduction of vacancies increases the lattice constant *a* and decreases *c*. The change in *a* is negligible, but a significant change in *c* is observed along the [0001] direction. The lattice constant changes for Ti_3_SiC_2_ are larger than those for Ti_3_AlC_2_. 

**Figure 7:**
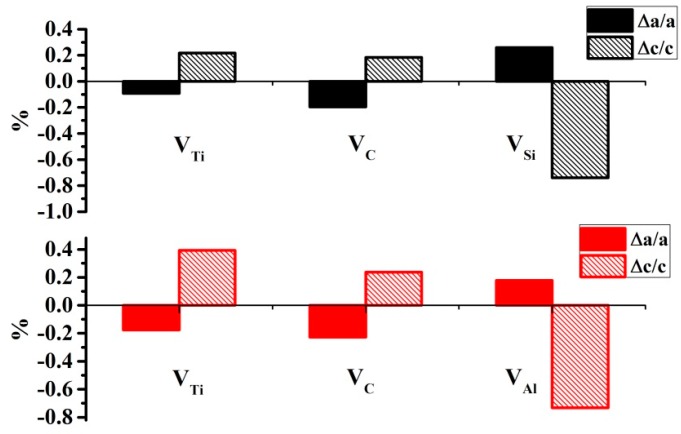
Lattice constant changes for Ti_3_SiC_2_ (black bar) and Ti_3_AlC_2_ (red bar) with respect to three types of on-site vacancies at a concentration of 12.5%. The difference between the results for Ti(a) and Ti(b) is very small, therefore the values are averaged as V_Ti_ for clarity.

The lattice constant changes induced by other types of vacancies were also calculated. The results for V_Ti_ and V_C_ are the opposite of those for V_A_. As shown in Figure 7, when V_Ti_ and V_C_ vacancies are introduced, the lattice constant *a* decreases and *c* increases. The effects of V_A_ and V_Ti_/V_C_ on the lattice constants differ because the interactions between the corresponding atoms and their surrounding atoms are different. In the formation of V_Ti_ and V_C_, the strong Ti–C covalent bond is broken; this is the driving force behind the decrease in the lattice constant in the (0001) plane. In the formation of V_A_, the bonds between Al/Si atoms and the surrounding atoms are broken. According to our previous analysis of diffusion barriers, the interactions between the Al/Si layer and the two neighboring Ti layers are stronger than the in-plane interactions for V_A_. The formation of V_A_ therefore contracts the materials along the [0001] direction. 

**Figure 8:**
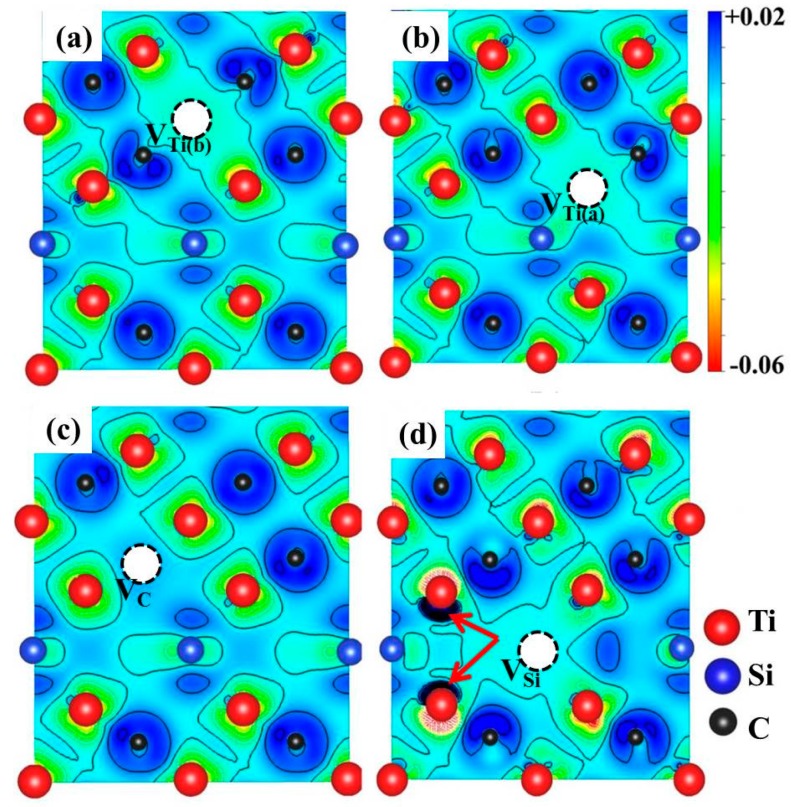
Deformation charge density (difference between crystal charge and atomic charge distribution) on (1, 1, $\overset{-}{2}$, 0) plane of Ti_3_SiC_2_ with (**a**) V_Ti(b)_; (**b**) V_Ti(a)_; (**c**) V_C_; and (**d**) V_Si_ vacancies. Contours added with intervals of 0.005 electrons/Bohr^3^. Red and blue isosurfaces correspond to electron-depleted and electron-enriched zones, respectively. White circles indicate positions of vacancies. Electron accumulation areas around two Ti atoms neighboring V_A_ in (d) are indicated by red arrows.

To verify this conclusion, the interactions between atoms in Ti_3_SiC_2_ with vacancies were analyzed based on the deformation charge densities. As shown in Figure 8, unlike the electron density distributions in the configurations of V_Ti_ and V_C_, there is an electron accumulation area around the two Ti atoms neighboring V_A_. The electron accumulation of these two Ti atoms along the [0001] direction indicates that the interaction between them is enhanced by the Si vacancy. The effects of V_Si_ on the lattice constants are therefore different from those of V_Ti_ and V_C_.

## 4. Conclusions

In this study, the properties of vacancies in Ti_3_AlC_2_ and Ti_3_SiC_2_, which are two of the most widely studied MAX phases, were investigated using first-principles calculations. Our results show that an A-group element vacancy (V_A_) has the lowest formation energy, therefore the vacancy–vacancy interactions, the effects of V_A_ on the lattice constants, and the charge redistribution of V_A_ were studied. The formation energy of V_Al_ is 0.9 eV lower than that of V_Si_, indicating that an A-group element mono-vacancy is more easily formed in Ti_3_AlC_2_. Although the diffusion barriers for Ti_3_SiC_2_ and Ti_3_AlC_2_ are similar (~0.95 eV), the vacancy properties are different. Our results show that the vacancy–vacancy interaction is attractive in Ti_3_AlC_2_ but repulsive in Ti_3_SiC_2_. The vacancies therefore tend to disperse in Ti_3_SiC_2_ but are accommodated in Ti_3_AlC_2_. Based on these results, we conclude that Ti_3_SiC_2_ should be more stable than Ti_3_AlC_2_ in a corrosive environment. The introduction of V_Ti_ and V_C_ vacancies causes the lattice constant *c* along the [0001] direction to increase for both Ti_3_SiC_2_ and Ti_3_AlC_2_. The changes in the lattice constants caused by V_A_ are opposite. The effect of V_A_ on the lattice constants is explained by enhanced interactions of nearby Ti layers.

## Figures and Tables

**Table 1:** Calculated (Cal.) lattice constants *a* and *c* (Å), *c/a* ratio, and internal structural parameters *z*_Ti_ and *z*_C_ for Ti_3_SiC_2_ and Ti_3_AlC_2_. Experimental values (Exp.) are also listed.

Material	Method	*a* (Å)	*c* (Å)	*c/a*	*z*_Ti_	*z*_C_
Ti_3_SiC_2_	Cal.	3.075	17.734	5.767	0.135	0.572
	Exp. [42]	3.07	17.67	5.76	0.135	0.568
Ti_3_AlC_2_	Cal.	3.082	18.642	0.648	0.127	0.569
	Exp. [6]	3.075	18.578	0.641	0.128	0.564

**Table 2:** Calculated elastic properties of Ti_3_SiC_2_, including the bulk modulus *B*, the shear modulus *G*, the Young’s modulus *E*, the Poisson ration *v*, the acoustic wave velocities (*V_l_*, *V_t_*, *V_a_*), and the Debye temperature *Θ*_D_. The experimental values [46] are also listed for comparison.

**Properties**	***B* (GPa)**	***G* (GPa)**	***E* (GPa)**	***v***
Cal.	200.3	132.3	325.2	0.23
Exp.	187	142	339	0.2
**Properties**	***V_l_* (Km/s)**	***V_t_* (Km/s)**	***V_a_* (Km/s)**	***Θ*_D_ (K)**
Cal.	9.17	5.43	6.0	780
Exp.	9.14	5.61	6.2	804

**Table 3:** Diffusion barriers for V_A_ in Ti_3_SiC_2_ and Ti_3_AlC_2_. Self-diffusion barriers of Al, C, and Ni are also listed for comparison.

Material	Ti_3_SiC_2_/Ti_3_AlC	Cu	Al	Ni
Barrier (eV)	0.95	0.92 [47]	0.61 [48]	1.4–1.8 [49,50]
